# Rapid Detection of Shrimp White Spot Syndrome Virus by Real Time, Isothermal Recombinase Polymerase Amplification Assay

**DOI:** 10.1371/journal.pone.0104667

**Published:** 2014-08-14

**Authors:** Xiaoming Xia, Yongxin Yu, Manfred Weidmann, Yingjie Pan, Shuling Yan, Yongjie Wang

**Affiliations:** 1 Laboratory of Quality and Safety Risk Assessment for Aquatic Products on Storage & Preservation, Ministry of Agriculture, Shanghai, China; 2 Shanghai Engineering Research Center of Aquatic-Product Processing & Preservation, Shanghai, China; 3 College of Food Science and Technology, Shanghai Ocean University, Shanghai, China; 4 Institute of Aquaculture, University of Stirling, Stirling, United Kingdom; 5 Institute of Biochemistry and Molecular Cell Biology, University of Göttingen, Göttingen, Germany; German Primate Center, Germany

## Abstract

White spot syndrome virus (WSSV) causes large economic losses to the shrimp aquaculture industry, and thus far there are no efficient therapeutic treatments available against this lethal virus. In this study, we present the development of a novel real time isothermal recombinase polymerase amplification (RPA) assay for WSSV detection on a small ESEQuant Tube Scanner device. The RPA sensitivity, specificity and rapidity were evaluated by using a plasmid standard as well as viral and shrimp genomic DNAs. Compared with qPCR, the RPA assay revealed more satisfactory performance. It reached a detection limit up to 10 molecules in 95% of cases as determined by probit analysis of 8 independent experiments within 6.41±0.17 min at 39°C. Consequently, this rapid RPA method has great application potential for field use or point of care diagnostics.

## Introduction

White spot syndrome virus (WSSV), belonging to the genus *Whispovirus* of the family *Nimaviridae* [1], is a highly infectious virus often causing white spots on the exoskeleton of shrimp and infects many crustaceans including shrimps, lobsters, freshwater crayfish and crabs [2]–[4]. Since WSSV was first detected in Taiwan in 1992 [5], it has spread worldwide and brought about large economic losses to the shrimp aquaculture industry [4]–[6].

Thus far, unfortunately, effective therapeutic methods are unavailable to block or reduce the outbreaks of WSSV. Hence, powerful surveillance management is crucial to monitor the WSSV spread and infection in brood-stocks, larvae and adult shrimps. It has been suggested that early diagnosis is one of the most efficient strategies to monitor the WSSV occurrence in shrimp farming facilities [7], [8].

In 2006, Piepenburg and coauthors reported a novel molecular diagnostic approach, recombinase polymerase amplification (RPA), which coupled isothermal recombinase-driven primer targeting the template with strand-displacement DNA synthesis [9]. RPA achieves exponential amplification with no need of pretreatment of the genomic DNA sample and is performed at constant low temperature (39–42°C) [9]. In RPA, recombinase UvsX with its co-factor UvsY combines with oligonucleotide primers to generate a recombinase-primer complex; the complex scans for reverse complementary sequences along a DNA template; strand displacement amplification starts with the presence of *Sau* polymerase (*Staphylococcus aureus*), once the duplex DNA of primer and template is formed. Based on a fluorescent-labeled probe detection system, the RPA assay is able to achieve a real time recording of the amplification event [10]–[12].

Here we present the development and application of a real time RPA assay for rapid detection of WSSV, the most lethal and contagious shrimp pathogen known to date, in order to pave the way for mobile/portable diagnostics of WSSV.

## Materials and Methods

### Viral and shrimp DNA samples

Genomic DNAs of WSSV and *Penaeus monodon* nudivirus (PmNV, the so-called Monodon baculovirus) were kindly provided by the Third Institute of Oceanography, State Oceanic Administration of China and National Taiwan University, respectively. Infectious hypodermal and hematopoietic necrosis virus (IHHNV) genomic DNA was extracted from diseased shrimps in this study. The shrimps were sampled with permission from an aquaculture farm in Shanghai, China.

### Preparation of plasmid standard

A 757 bp of WSSV DNA fragment containing *vp28* was amplified for preparation of a plasmid standard. The 50 µl PCR contained 22 ng WSSV DNA, 10 µM of each primer (Table 1), and 25 µl 2×*Taq*mix (Tiangen, Beijing, China). The thermal cycle program was as follows: 94°C for 4 min, followed by 30 cycles of 94°C for 30 s, 60°C for 30 s, 72°C for 60 s and a final extension step of 72°C for 10 min. Amplification products were checked by 1.5% agarose gel electrophoresis. The target bands of the amplification products were excised from the gel and purified using QIAquickGel extraction kit (Qiagen, Germany) according to the manufacturer’s instruction. The purified amplicons were ligated into the pGM-T vector using the pGM-T Cloning Kit (Tiangen, Beijing, China). The recombinant plasmid was transferred into competent *Escherichia coli* TOP10 cells (Tiangen, Beijing, China) following the manufacturer’s instructions. Recombinant plasmids were identified by colony PCR. The correct insert was verified by sequencing and quantified by using the Quant-iT PicoGreen dsDNA Reagent and Kits (Invitrogen, USA).

**Table 1:** PCR primers used in this study.

Name	Sequence 5′–3′	Genomelocation(AF332093)	Productsize (bp)	Reference
WSSVPCR-757 FP	TTGCCAATTGTCCTGTTACGTACTCTG	244110–244136		
WSSVPCR-757 RP	ACGATTTATTTACTCGGTCTCAGTGCC	244866–244840	757	This study
VP28–140F	AGGTGTGGAACAACACATCAAG	244610–244631		
VP28–140R	TGCCAACTT CATCCTCATCA	244750–244731	140	[28]

WSSV PCR-757 FP and RP were used for the PCR-cloning of *vp28*.

VP28–140F and R were used in qPCR assay.

The concentration of the recombinant plasmid was converted to copy numbers based on the following equation: Number of copies = (M×6.02×10^23^×10^−9^)/(n×660), the M is the amount of DNA in nanogram, n is the number of the recombinant plasmid in base pair, and the average weight of one base pair is assumed to be 660 Da. The prepared plasmid standard was aliquoted and stored at −80°C before use.

### qPCR

The recombinant plasmid standard was serially diluted to a range from 5 to 5×10^6^ copies per microliter. Two µl of each dilution were utilized for qPCR assay on an ABI 7500 Fast qPCR system with 2.0.1 version software (Applied Biosystems, USA). The reaction mix (20 µl) contained 2 µl genomic DNA, 0.6 µM of primers VP28–140F and VP28–140R (Table 1), and 10 µl Maxima SYBR Green qPCR Master Mix (2×) (Fermentas, MBI). The thermal cycle program was described as follows: 95°C for 10 min, followed by 40 cycles of 94°C for 15 s, 60°C for 60 s.

### RPA

The RPA primers were designed for the *vp28* gene by a multiple sequence alignment of 48 nucleotide sequences available in GenBank. In reference to WSSV genomic sequence AF332093.2 (ORF421, 244243–244858 bp), the RPA amplicon was placed between position 244,384 and 244,507 (length 124 bp). A probe was designed for real time RPA assay. It contained a tetrahydrofuran (THF) flanked by a dT-Fluorophore and dT-Quencher group. Thirty-one and seventeen nucleotides were located 5′ and 3′ to the THF site, respectively (Table 2).

**Table 2:** RPA primers and probe designed in this study.

Name	Sequence 5′–3′	Genomelocation(AF332093)	Productsize (bp)
WSSV RPA FP	CATGGATGAAAACCTCCGCATTCCTGTGAC	244384–244413	
WSSV RPA RP	CATCAGACTTTCCATTGCGGATCTTGATTTTG	244507–244476	
WSSV RPA P	TGCTGAGGTTGGATCAGGCTACTTCAAGA(BHQ1-dT)G(THF)C(FAM-dT)GATGTGTCCTTTGAC (phosphate)	244414–244462	124

WSSV RPA FP and RP: RPA primer, WSSV RPA P: RPA exo probe, BHQ1-dT: thymidine nucleotide carrying Blackhole quencher1, THF: tetrahydrofuran spacer, FAM-dT: thymidine nucleotide carrying Fluorescein, phosphate: block elongation.

RPA was performed at 39°C for 20 minutes in a 50 µl volume containing 420 nM of each primer, 120 nM probe, 14 mM Mg acetate, enzymes and 1×rehydration buffer (TwistAmp exo kit, TwistDX, Cambridge, UK), and 2 µl recombinant plasmid standard (1 µl for 5 copies dilution) or viral or shrimp DNA. An ESEQuant Tube Scanner device (Qiagen Lake Constance, Stockach, Germany) was used to detect the fluorescence signals.

### Determination of sensitivity and specificity

The analytical sensitivity of RPA was tested using the WSSV quantitative plasmid standard in a range of 1000, 100, 10 and 5 copies per reaction for 8 independent assays. The threshold time was plotted against the log number of the detected molecules and a semi-log regression was calculated. The probit regression was calculated using the IBM SPSS Statistics 20.0 (IBM, New York, USA).

The specificity of the WSSV RPA assay was tested by using the genomic DNA extracted from the white leg shrimp (*Litopenaeus vannamei*), giant black tiger prawn (*Penaeus monodon*), Chinese mitten crab (*Eriocheir sinensis*), human, IHHNV, and PmNV.

### Detection of shrimp samples

Forty-four shrimp samples with or without WSSV infection were used to test the performance of the RPA and qPCR assays. DNA extraction was performed by using the TIANamp Marine Animals DNA Kit (Tiangen, Beijing, China). The assays were carried out as described above.

### Statistic analysis

Values were represented as the mean with standard deviation (SD). Statistical analysis was performed using the IBM SPSS Statistics 20.0 (IBM, New York, USA).

## Results

### qPCR sensitivity

**Figure 1:**
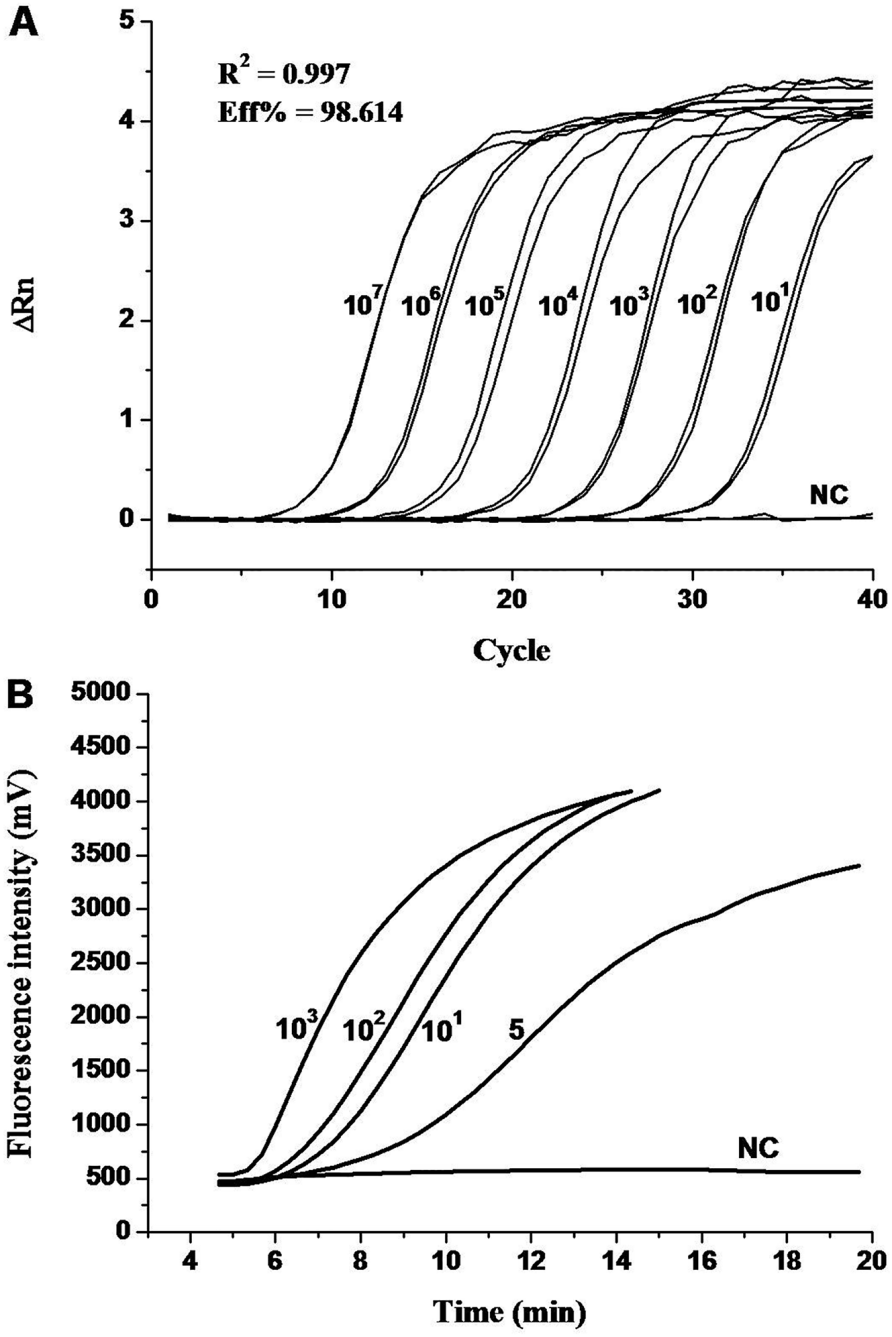
**(A) Amplification curve of qPCR** showing 10-fold serial dilution of the WSSV DNA standard plasmids (10^7^ to 10 copies per reaction). **(B) Amplification curve of real time RPA** showing a dilution ranges of 10^3^, 10^2^, 10, 5 molecules of the WSSV DNA standard plasmids (Graph generated by ESEquant tubescanner software). NC refers to negative control.

**Figure 2:**
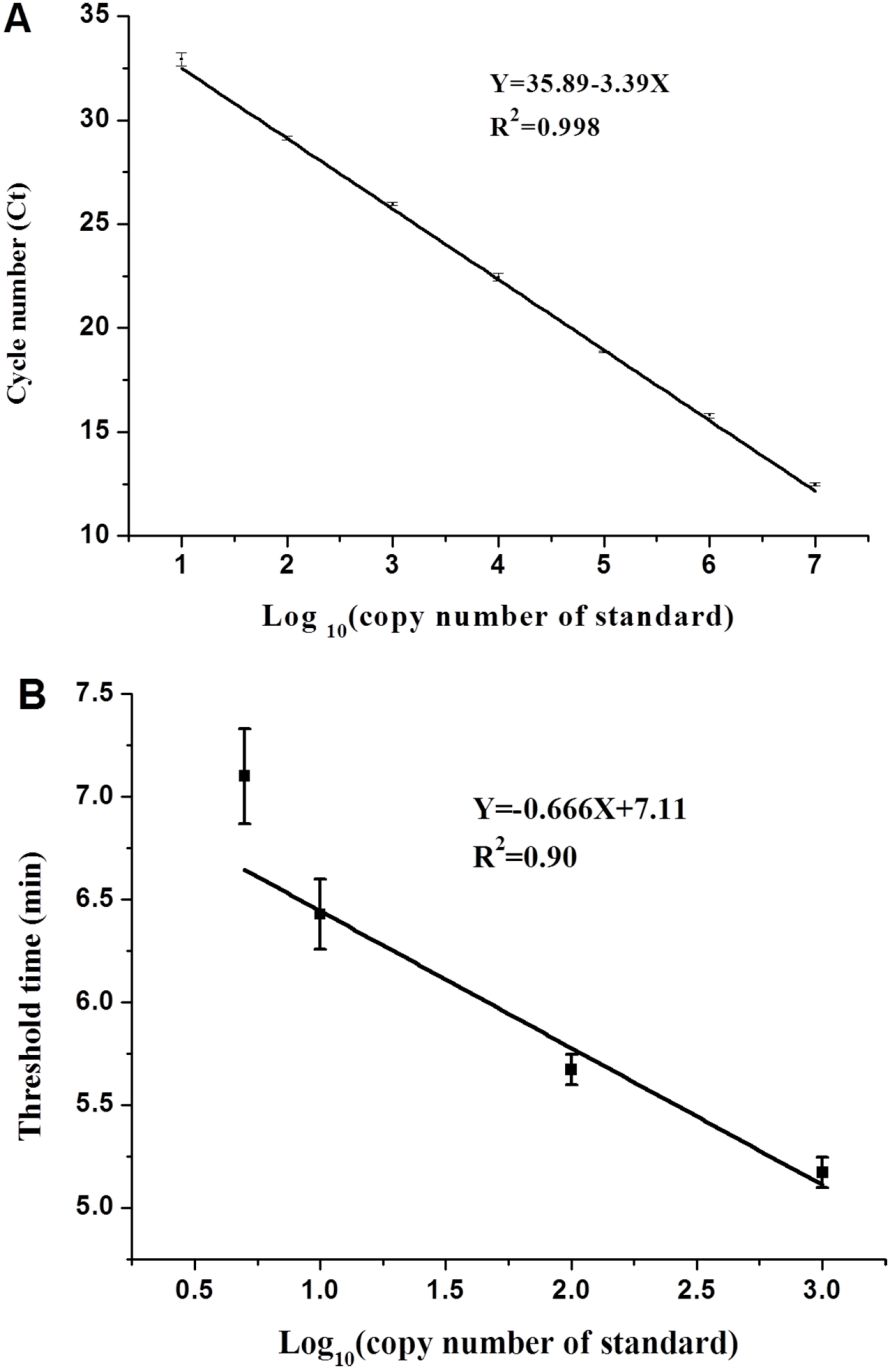
**(A) The reproducibility of WSSV qPCR assay** (C_T_ values were represented as the mean ± standard deviation (SD)). Standard regression line was generated based on two data sets. **(B) The reproducibility of WSSV real-time RPA assay** (Threshold time was represented as the mean ± standard deviation (SD)). Standard regression line was generated based on eight data sets.

The qPCR assay revealed a high amplification efficiency (Eff% = 98.464) as well as a strong linear correlation (R^2^ = 0.9973) between the threshold cycles (Ct) of the plasmid standards ranging from 1.0×10^7^ to 1.0×10^1^ copies (Figs.1 and 2). The highest detection sensitivity of the qPCR was 10 molecules. Two independent repeats confirmed the reproducibility of the performance of the qPCR (Figs.1 and 2). Negative controls did not show any amplification signals for each run (Fig. 1).

### RPA sensitivity, rapidity and reliability

**Figure 3:**
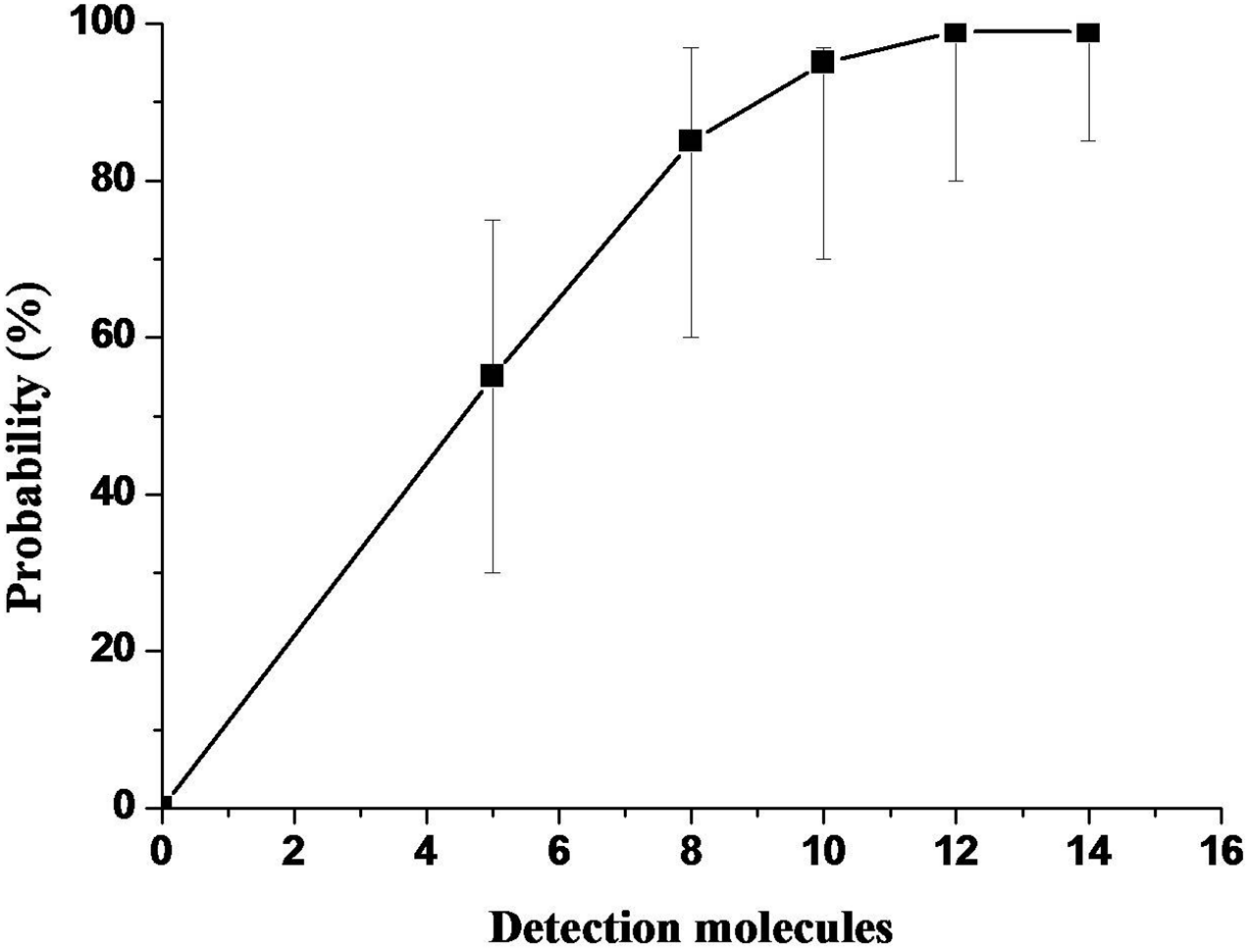
Probit regression of WSSV real-time RPA using the data of 8 runs. (The sensitivity of 10 molecules with 95% reliability by SPSS probit regression analysis).

To ascertain the detection limit, the RPA assay was firstly performed using the prepared plasmid standard of 10^7^ to 10 copies. The high concentration (10^7^−10^5^ copies/µl) of standards showed extremely high amplification signals in just 3–4 min of reaction (data not show). Accordingly, it was unnecessary to include those high concentrations of standard for evaluation of the detection sensitivity of the RPA assay; only the standard concentrations of 10^3^, 10^2^, 10, and 5 copies/µl were used as templates in the subsequent RPA assay. As a result, a detection limit of up to 5 copies detected within 7.12±0.50 min was achieved (Figs. 1 and 2). By applying the data analysis software Origin, the standard deviation of the threshold time values for 10^3^ down to 5 copies ranged from 0.11 to 0.50 minutes. The R square value of the standard regression line was 0.90 (Fig. 2). The detection rate of 10 and 5 copies was 87.5% and 75.0%, respectively, as determined by 8 independent experiments. Probit analysis of the reliability of the RPA using the results of 8 independent assays revealed that in 95% of cases a minimum of 10 molecules are detected, and the detection rate ranged from 70 to 99% (Fig. 3).

### RPA specificity

The specificity of RPA assay was confirmed by cross detection assays using the genomic DNA of the shrimp, crab, human, IHHNV, and PmNV. None of these were amplified.

### Assay performance on shrimp samples

**Figure 4:**
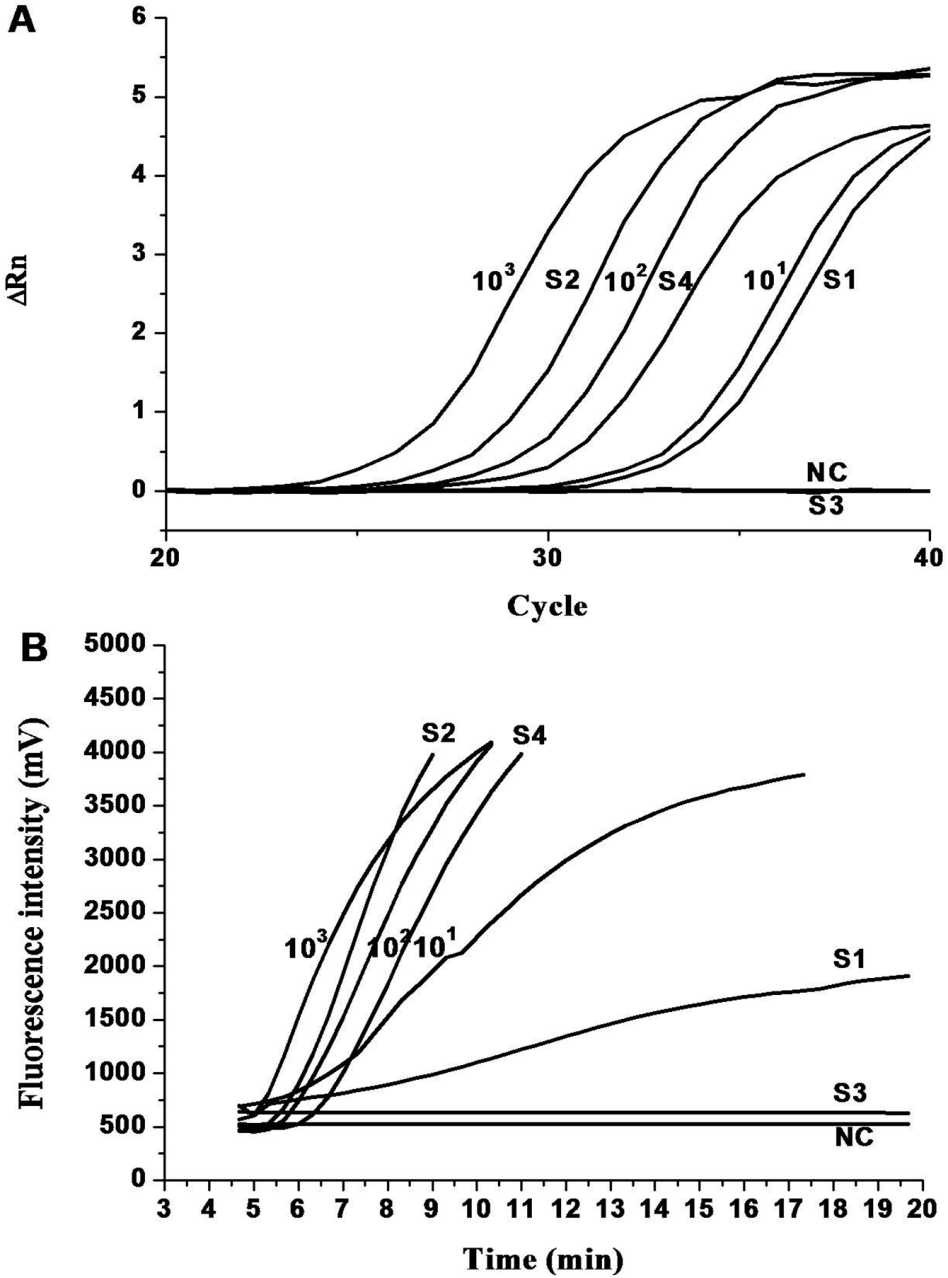
**Amplification curve of qPCR (A) and real-time RPA (B).**, showing 10-fold serial dilutions of standard plasmids and 4 out of 44 representative shrimp samples. Numbers (10^3^ to 10) near black lines: *vp28* copies/reaction, S1–S4 near black lines: 4 representative shrimp samples, NC: negative control.

Ten shrimp individuals were detected originally by using both qPCR and RPA. Eight shrimps were WSSV positive, and two negative (Fig. 4). Both assays showed a similar performance regarding the analytical sensitivity (Fig. 4). As for the RPA assay, the WSSV copy number was between 1000 and 100 for sample 2, between 100 and 10 for sample 4, and less than 10 for sample 1. These results were in agreement with the calculated WSSV copy number of 272 (sample 2), 51 (sample 4) and 7 (sample 1) obtained by using qPCR (Fig. 4). It indicated that RPA was able to detect as few as 10 WSSV particles in the shrimp samples. In addition, the threshold times of samples 2, 4 and 1 were 6.1, 7.0, and 9.0 min in RPA, respectively; by contrast, the detection C_T_ value of the qPCR was approximately 28.9, 31.4 and 34.4, respectively (Fig. 4).

To further confirm the stability of the RPA assay, 34 more shrimp individuals were subject to the detection for the infection of WSSV. In both RPA and qPCR assays, as a result, 22 shrimps were WSSV positive, and 11 negative. Only one sample showed week positive amplification signal in RPA assay, but negative in qPCR. Accordingly, the WSSV-RPA assay turned out to be reliable.

## Discussion

WSSV is one of the most serious pathogens in the shrimp-farming industry [13]–[15]. Early and rapid monitoring its infection with high sensitivity represents the most efficient strategy in the control and prevention of the outbreaks of its diseases [8].

### The limits of other detection methods

Thus far, a variety of methods have been developed to detect WSSV, which can be divided into two groups based on their targeting molecule of protein or DNA. The first group is antibody-based immunological methods, which are generally simple and low-cost detection systems with considerable rapidity [16]–[21]. However, these detection techniques encounter the problems of both low sensitivity and limited throughput. The other group comprises DNA-based detection methods (Table 3). Conventional PCR is widely used [22], [23], which is the powerful method to detect pathogens in different kinds of hosts, such as plants, animals, or humans, in laboratory [24], [25]. In spite of its superiority of sensitivity, the natures of PCR, such as the requirement of expensive thermal cycler, time-consuming, labor-intensive, especially depending on the classical agarose gel electrophoresis to read amplification results, greatly impede its application *in situ*. Improved PCR methods developed based on the conventional PCR, including qPCR [26]–[28], nested PCR [29] and insulated isothermal PCR [30], [31], are not feasible for pond-side detection either and incur high costs for routine monitoring of WSSV during shrimp cultivation. Although isothermal LAMP [32]–[34], detects WSSV using a simple heating block, the amplification products are not amenable for quantification. Additionally, the design of appropriate LAMP primers is more complicated than that for PCR and RPA.

**Table 3:** Performance of various methods used for detecting WSSV DNA.

Method	Target gene	Reactiontemperature (°C)	Reaction time(min/C_T_)	Sensitivity	Reference
qPCR (SYBR green)	*vp28*	60–94	C_T_ = 30	12 copies/reaction	[28]
qPCR (TaqMan probe)	*vp28*	60–94	C_T_ = 41	5.9 copies/reaction	[26]
Insulated Isothermal PCR(TaqMan probe)	*wsv360*	94	30	10 copies	[30]
Nest PCR	ORF119	54–94	137	0.015 pg	[29]
PCR	*vp28*	60–94	40	100 pg	[22]
Real-time LAMP (lateralflow dipstick)	ORF191	65	50–60	100 copies	[32]
LAMP (fluorescenceenergy transfer-basedprobes)	ORF191	65	60	100 copies	[33]
RPA	*vp28*	39	7	5 copies/reaction	This study

### The advantages of RPA compared to qPCR regarding to temperature, sensitivity and reaction time

Recently, Mendoza-Cano and coauthor [28] reported a SYBR green-based qPCR assay for the detection of WSSV, which revealed a high sensitivity of 12 copies per sample. In order to evaluate the RPA assay, we also established this published qPCR assay in this study, which showed a slightly higher sensitivity of 10 copies per reaction compared to that of [28].

As expected, the RPA method established in this study revealed a satisfactory performance for the detecting of shrimp WSSV DNA. The isothermal RPA was performed at 39°C, while the two-step thermal cycling qPCR had to be conducted at 94 to 60°C. The low and constant reaction temperature of RPA paves the way for simpler devices and for mobile pond-site and point-of-care detection of WSSV.

Besides, the detection limit of RPA is as few as 5 copies of WSSV DNA per reaction, which is comparable to that of qPCR. The reaction time of RPA was less than 10 min; by contrast, approximately 33 cycles (134 minutes) were required in the qPCR assay in order to achieve the similar detection sensitivity as in RPA. Obviously, the RPA assay saves almost 100 min of reaction time in comparison with the qPCR. This reduction in assay time makes the WSSV-RPA assay a handy tool for monitoring WSSV infection during shrimp cultivation on short notice in a real time strategy.

### The *vp28* as the target gene

Most of the methods for detecting WSSV target *vp19* [35], *vp28* [22], [26], [28], *wsv360* [30], ORF191 [32], [33] and ORF 36 [36]. Among these genes, the *vp28* is mostly widely used. VP28 is the most abundant envelope protein in WSSV and plays an essential role in viral attachment during early events of virus infection [37], [38]. The *vp28* gene detection assay appears to bear much higher sensitivity in comparison to the methods targeting other genes (Table 3), and there are plenty of sequences of *vp28* gene available in GenBank, which contributes to the design of high-coverage primers and probes. In addition, sequence alignment analysis indicated that *vp28* is a highly conserved gene in WSSV (Data not shown). Therefore, the diagnostic methods developed based on *vp28* is feasible for universal detection of various WSSV isolates.

### The convenience of application of RPA to *in situ* diagnostics

The commercially available TwistAmp exo kit (TwistDX, Cambridge, UK) contains buffer and reaction mixtures, which include enzymes and nucleotides, all provided in a dried pellets. Just primers, probe, and DNA template need to be added. Again this recommends RPA is a promising technique designed for point-of-care or field diagnoses.

Importantly, it is necessary to point out that the size of the ESEquant Tube Scanner, the reaction signal recording device, is just 17.4 by 18.8 cm with a weight of about 1 kg (including the laptop). It means the Tube Scanner device is significantly light, small and convenient for point of care monitoring [40]. Besides, the Tube Scanner is much cheaper than any of the mobile PCR devices. Therefore, RPA implemented on a tubescanner is a very promising nucleic acid detection method that could easily be applied on shrimp farms at much lower investment than for using mobile qPCR on real time cyclers.

Recently, Lutz and coauthors combined RPA with a foil based microfluidic LabDisc system [39]. This improvement together with the development of automatic sample preparation system could eventually lead to the development of RPA panels or chips for the simultaneous detection of different categories of infectious agents, e.g., syndromic panels of bacteria, and DNA and RNA viruses, in small point of care devices.

In conclusion, with the assistance of the mobile device (ESEquant Tubescanner system), we have developed an extremely efficient and highly sensitive isothermal real time RPA assay for the detection of WSSV. The WSSV real time RPA assay appears to be much more suitable for mobile testing on pond sites. More work, such as validation of specificity and reproducibility, needs to be done to apply the established WSSV-RPA assay to the diagnostics.
